# Dietary Polyphenols, Berries, and Age-Related Bone Loss: A Review Based on Human, Animal, and Cell Studies

**DOI:** 10.3390/antiox3010144

**Published:** 2014-03-11

**Authors:** Patrice A. Hubert, Sang Gil Lee, Sun-Kyeong Lee, Ock K. Chun

**Affiliations:** 1 Department of Nutritional Sciences, University of Connecticut, Storrs, CT 06269-4017, USA; E-Mails: patrice.hubert@uconn.edu (P.A.H.); sanggil.lee@uconn.edu (S.G.L.); 2 Center on Aging, University of Connecticut Health Center, Farmington, CT 06030-5215, USA; E-Mail: klee@nso2.uchc.edu

**Keywords:** aging, bone loss, osteoporosis, oxidative stress, berries, polyphenols, anthocyanins

## Abstract

Bone loss during aging has become an increasing public health concern as average life expectancy has increased. One of the most prevalent forms of age-related bone disease today is osteoporosis in which the body slows down bone formation and existing bone is increasingly being resorbed by the body to maintain the calcium balance. Some causes of this bone loss can be attributed to dysregulation of osteoblast and osteoclast activity mediated by increased oxidative stress through the aging process. Due to certain serious adverse effects of the currently available therapeutic agents that limit their efficacy, complementary and alternative medicine (CAM) has garnered interest as a natural means for the prevention of this debilitating disease. Natural antioxidant supplementation, a type of CAM, has been researched to aid in reducing bone loss caused by oxidative stress. Naturally occurring polyphenols, such as anthocyanins rich in berries, are known to have anti-oxidative properties. Several studies have been reviewed to determine the impact polyphenol intake—particularly that of berries—has on bone health. Studies reveal a positive association of high berry intake and higher bone mass, implicating berries as possible inexpensive alternatives in reducing the risk of age related bone loss.

## 1. Introduction

Age-related bone disease has become a public health concern as average life expectancy has increased. One of the most prevalent forms of age-related bone disease today is osteoporosis, which is estimated to affect over 200 million individuals worldwide [1]. Osteoporosis currently affects 10 million Americans [2] with an expected increase to 14 million by 2020 [3]. Osteoporosis is defined as a decrease in bone mineral density (BMD), including calcium content [4]. In osteoporosis, the body slows down bone formation and existing bone is increasingly being resorbed to maintain the calcium balance [4]. Complications of bone loss include increased susceptibility to falls, fragile bones, decreased quality of life, and increased risk of mortality [4]. Many efforts are being made at identifying those at risk and decreasing their susceptibility [5]. These complications carry an economic burden of $17–$20 billion annually and are anticipated to reach $25 billion by 2025 [2,6]. With this projected increase, it is beneficial to identify the causes of osteoporosis to discover ways to prevent and/or treat them. 

The causes of osteoporosis are diverse and complex. Major age-related risk factors for osteoporosis include hormonal imbalance resulting in osteoclast and osteoblast dysfunction, increased oxidative stress, and chronic inflammation [7]. In women, the most prominent hormonal imbalance associated with age-related bone loss is the decrease in circulating estrogen levels following menopause. Estrogen deficiency has been shown to contribute to increased levels of reactive oxygen species (ROS) followed by increased proosteoclastogenic cytokine secretion from bone marrow cells [8]. Evidence has also demonstrated the inhibitory role of estrogen in osteoclast differentiation [9]. Generation of oxidative stress and chronic inflammation associated with aging can lead to excess bone resorption, causing osteoporosis [7,10]. Zhang *et al.* determined an association between age-related bone loss and oxidative stress by measuring advanced oxidation protein products (AOPP), malondialdehyde (MDA) and superoxide dismutase (SOD) in the femurs of young, adult, and old rats [11]. Levels of AOPP and MDA increased with aging, while SOD activity decreased. Femur BMD was significantly lower in the adult groups when compared with the young group, positively correlating with SOD activity, suggesting oxidative stress induced age related bone loss [11]. 

The Food and Drug Administration has approved several drugs called antiresorptive medications, such as bisphosphonates, hormone replacements, the selective estrogen receptor modulators (SERM) raloxifene and denosumab (anti-RANKL antibody) for the prevention and treatment of osteoporosis [12,13,14]. However, some of these agents have adverse effects that limit their efficacy and underscore the urgent need for new treatment/prevention options. Hormone replacement therapy was associated with an increased risk for stroke incidence and other heart conditions [14]. Adverse effects of bisphosphonates include renal impairment, hypocalcemia, hypophosphotemia, influenza-like illnesses, gastrointestinal distress, and musculoskeletal pain [15]. For this reason, complementary and alternative medicine (CAM) has garnered interest as a natural means of disease prevention. Natural antioxidant supplementation, a type of CAM, has been researched to aid in reducing bone loss caused by oxidative stress [16]. 

Antioxidant-rich foods may represent one strategy for slowing down age-related bone loss and improving bone remodeling. Dietary polyphenols have been associated with bone health, which may be in part attributable to their antioxidant capacity [17]. Several studies have identified greater fruit intake with decreased fracture risk, greater BMD, and decreased bone turnover [18,19]. As a natural source of antioxidants, berries have demonstrated health benefits, including anti-inflammatory and antioxidant properties [20]. Berries contain various polyphenols, the most prominent of which being anthocyanins. Anthocyanins are water-soluble glycosides belonging to the flavonoid family. Anthocyanins are commonly referred to as anthocyanidins, and comprise a variety of compounds including pelargonidin, cyanidin, delphinidin, peonidin, petunidin, malvidin [21]. Anthocyanins are one of the most effective phenolic compounds in anti-oxidative power [22]. Foods rich in anthocyanins have been associated with a reduced risk of cardiovascular disease, hyperglycemia, some types of cancer protection, and bone health [23]. However, limited research has been done with natural dietary polyphenols in berries and evidence from epidemiologic and animal studies is inconsistent. Furthermore, the mechanisms by which these polyphenols directly modify bone remodeling are not fully understood. This article provides a review of studies on age-related bone loss in relation to dietary polyphenols from berries to explain the underlying mechanisms of prevention of age-associated bone loss in cell, animal, and human studies.

## 2. Article Selection Criteria

Studies for this review were searched using the U.S. National Library of Medicine National Institute of Health online database PubMed. Keywords used to search for articles included bone, bone loss, oxidative stress, berries, anthocyanin, polyphenols, osteoblasts, osteoclasts, dietary behavior, and bone health. Articles were selected based on the three sections of the review. To discuss the actions of oxidative stress on bone health, articles were selected based on how well they delineate the subject matter. Included were cohort and cell mechanistic studies. For the remaining two sections, studies were included based on the strength of evidence provided and if a positive effect on bone was shown. Studies were excluded if supplementation included anything other than polyphenols that have known effect on bone health. For example, a study with supplementation of both calcium and polyphenol would be excluded. Since there were limited studies available portraying the direct effect of berries on bone, many studies were included demonstrating the anti-oxidative power of different berries on oxidative stress and inflammation to help apply that action to bone health. 

## 3. Underlying Mechanisms: Aging, Oxidative Stress, and Bone Turnover

Bone turnover is primarily regulated by osteoblasts and osteoclasts; osteoblasts are responsible for bone formation, while osteoclasts regulate bone resorption [7]. During the process of aging, the balance between bone resorption and formation is tipped towards enhanced resorption and decreased formation causing an overall loss of bone [24]. As the body ages, its skeleton becomes more susceptible to oxidative stress damage [24,25]. Oxidative stress is the result of an abundance of ROS with an insufficient antioxidant defense system affecting osteoblast and osteoclast regulation [26,27]. Osteoclast differentiation and activity are stimulated through binding between receptor activator of nuclear factor kappa-B ligand (RANKL) from osteoblasts/stromal cells and receptor activator of nuclear factor kappa-B (RANK) on osteoclast precursors in the presence of macrophage colony stimulating factor (M-CSF). Binding of RANK and RANKL induces signal transduction through the nuclear factor kappa B (NF-κB) pathway and activator protein-1 (AP-1) pathway initiating the differentiation of osteoclast precursor cells into preosteoclasts. It has been also recognized that several inflammatory cytokines including interleukin (IL)-1, tumor necrosis factor-alpha (TNF-α), and IL-6 can promote the production of RANKL by osteoblasts indicating that chronic inflammation during aging may cause bone loss. In addition, TNF-α can intensify osteoclast activity by integrating with RANKL-induced signal transduction pathways. ROS acts as a mediator of RANKL signaling of osteoclastogenesis and osteoclast lifespan; RANKL promotes the lifespan of osteoclasts, indicating that ROS also plays a role in this extension [28]. Garret *et al.* found that when ROS were generated next to bone surfaces, it caused the differentiation of osteoclasts stimulating bone resorption [29]. An *in vivo* study showed that osteoclast number and resorption activity were simultaneously increased between bone and bone marrow under increased oxidative stress [29]. The treatment of oxides, hydrogen peroxide and xanthine oxidase to bone cells increased oxidative stress and inhibited osteoblastic cell differentiation [30]. Oxidative stress can inhibit osteoblastic differentiation through the mechanistic action of extracellular signal-regulated kinases (ERK) and ERK-dependent NF-κB signaling pathways [31].

Oxidative stress markers measured in the plasma of an elderly population with and without osteoporosis revealed an association between antioxidant status, oxidative stress, and osteoporosis risk. In the group with osteoporosis, glutathione peroxidase (GPx) activity was significantly decreased along with a higher frequency of oxidative stress and decreased BMD. SOD was slightly decreased as well, not to a level of significance, but the SOD/GPx activity ratio was significantly increased [26]. SOD is normally decreased in osteoporotic patients [32,33]. The higher ratio seen may be in relation to the differences in SOD and GPx activities between the two groups. The results indicate a greater amount of oxidative stress in osteoporotic patients along with overall lower antioxidant activity and decreased BMD [26]. In a study performed to determine how ROS plays a role in bone metabolism, a control group was compared to groups with osteoporosis, osteodystrophy, and bone fractures. Blood serum samples were collected measuring markers of bone formation, oxidative stress, and antioxidant activity. Supporting the earlier study results, decreased serum GPx was also seen in those subjects with osteoporosis and bone fractures [34].

Decreased blood antioxidant activity and higher oxidative stress markers are associated with aging and decreased bone mass. Normally, the body is able to prevent excessive production of ROS through the body’s natural antioxidant defense system by producing enzymes such as SOD, catalase, and glutathione [25,27]. Mice that are SOD deficient exhibited lower bone mass, lower osteoblast and osteoclast numbers with decreased RANKL expression, and higher ROS levels compared with wild type mice [35]. When oxidative stress is introduced, an increase in osteoclast number is normally expected. However, in this study, osteoclast number decreased which may be indicative of the body’s decreased ability at bone remodeling. This is naturally expected in the aging process. Administration of vitamin C to those SOD deficient mice normalized bone mass, suppressed ROS levels and increased the lifespan of osteoblasts, indicating the possible impact antioxidant supplementation may have on oxidative stress related bone loss [35].

## 4. Polyphenol Intake and Bone Health

Fruits and vegetables, or other plant sources have been suggested to have a possible bone protection mechanism [36,37]. Many studies have reported positive associations between greater fruit and vegetable intake and bone health [38]. Table 1 summarizes epidemiological studies on dietary polyphenols and bone turnover. New *et al.* reported that higher BMD in the femoral neck among woman was positively associated with high dietary intake of fruit in childhood [39]. Diets high in fruits, vegetables, and whole grains, and low in fat were associated with a reduced risk of falls and fractures and greater BMD in men and women [40,41]. Habitually high dietary flavonoid intakes are associated with an increase in BMD [42]. Dried plum, ranked one of the highest in antioxidant value, was associated with an increase in insulin-like growth factor-1 (IGF-1) and alkaline phosphatase activity (ALP), both markers of bone formation, and BMD [43,44]. 

**Table 1:** Summary of human studies on dietary polyphenols and bone turnover.

First author, year [ref.]	Study Design	Measurements/Treatments	Results
Hardcastle *et al.*, 2011 [19]	Observational Study, premenopausal women (*n* = 2929)	Diets via FFQ analyzed for flavonoid intake Measured BMD at femoral neck and lumbar spine	Catechin and procyanidin associated with increased BMD Flavanones showed no effect
Welch *et al.*, 2013 [42]	Observational Study, women (twins) (*n* = 3160)	Habitual intakes of flavonoids and subclasses via FFQ; bone density	Anthocyanins associated with highest observed BMD High flavanone intake positively associated with hip BMD
New *et al.*, 2000 [39]	Cross sectional, healthy women 45–55 years (*n* = 62)	BMD at lumbar spine and femoral neck; bone resorption, nutrient intakes via FFQ	Femoral neck BMD higher in women who consumed high amounts of fruit in childhood
Langestmo *et al.*, 2011 [40]	Retrospective Cohort Study, random selection (*n* = 5188)	Assessed dietary patterns using FFQ to determine low-trauma fracture	Nutrient dense (fruits, vegetables, whole grains) associated with reduced risk of fracture
McTiernan *et al.*, 2009 [41]	Randomized Controlled Trial, Postmenopausal women (*n* = 29,294)	Intervention: Women’s Health Initiative Dietary Modification (low fat, increase fruit and vegetable for 8 years; Assessed fracture falls, BMD)	Intervention associated with lower rate of falls No effect on BMD
Arjmandi *et al.*, 2002 [44]	Randomized Controlled Trial Postmenopausal osteopenic women (*n* = 48)	Two groups; Intervention: dried plum (100 g/day) *vs.* comparative dried apples (75 g/day) for 3 months	Dried plum increased IGF-1, ALP, and BAP
Hooshmand *et al.*, 2011 [43]	Randomized Controlled Trial, postmenopausal osteopenic women (*n* = 160)	Two groups; Intervention: dried plum (100 g/day) *vs.* comparative dried apples for 12 months	Dried plum increased BMD, decreased serum BAP and TRAP-5b

BMD: bone mineral density; FFQ: food frequency questionnaires; BAP: bone specific alkaline phosphatase; TRAP-5b: tartrate resistant acid phosphatase-5b; IGF-1: insulin like growth factor-1; ALP: alkaline phosphotase.

Table 2 summarizes animal and cell studies involving polyphenol and bone turnover. In ovariectomized (OVX) mice, supplemented with different concentrations of dried plum, femur and tibia bone mass was increased [45,46,47,48]*.* Bone loss is not limited to loss of estrogen exhibited in OVX mice. Orchidectomized (ORX) mice exhibited decreases in bone mass after procedure and when supplemented with dried plums demonstrated an increase in BMD [47].

Berries may demonstrate a positive effect on bone metabolism due to their antioxidant power by phenolic content [49]. Various studies have reported that berry intake can increase antioxidant status and reduce the level of inflammatory biomarkers *in vivo*. Cranberry juice significantly increased plasma antioxidant capacity, red blood cell (RBC) oxidative resistance, and SOD in OVX rats [50]*.* Bilberry was shown to decrease MDA levels, and decrease IL-6 and IL-15 (inflammatory mediators) in oxidative stress induced states [51,52]. Bilberry and black currant anthocyanins supplemented together suppressed NF-κB activation in human monocytes [53]. Chokeberry supplemented in mice increased catalase and GPx activity in hepatic cells [54]. Lingonberry increased SOD and glutathione reductase activity while increasing the concentration of reduced glutathione *in vivo* [55]. Based on these results, berries, such as blueberry and cranberry, may have a profound impact on bone due to their phenolic content. Blueberry was shown to increase whole body BMD and serum ALP in OVX rats [56]. Anthocyanins have also been shown to reduce oxidative damage and inflammation, and increase BMD [42,57]. In the review by Shen *et al.* [18], berries were found to prevent deterioration of whole body BMD, and prevent bone turnover due to the anti-oxidative and anti-inflammatory properties of anthocyanins. Studies from the review are summarized in Table 3. Feeding rats a blueberry diet early in life was shown to prevent OVX induced bone loss in adult life [58]. However, cranberry juice supplementation demonstrated no effect on bone health in ORX mice [50].

**Table 2:** Summary of animal and cell studies on dietary polyphenols and bone turnover.

First author, year [ref.]	Study Design	Measurements/Treatments	Results
Bu *et al.*, 2009 [59]	MC3T3-E1 cells (mouse osteoblast cell line)	Treated with 0, 2.5, 5, 10 and 20 µg/mL doses of dried plum extract and TNF-α	Increased intracellular ALP activity Increased expression of IGF-1 Stimulated mRNA expression of osteoblast marker genes (Runx2, osterix)
Bu *et al.*, 2008 [60]	RAW 264.7 (murine macrophage) and primary bone marrow cells	Treated with 10, 20, and 30 µg/mL dried plum extracts for 4 days	Decreased osteoclast differentiation Downregulated osteoclast precursor cyclooxygenase expression and nitric oxide TNF-α expression decreased over time
Deyhim *et al.*, 2005 [45]	SHAM and OVX 3 month old female rats	6 groups: Sham Control OVX-Control OVX + 17 Beta-estradiol OVX + 5% Dried Plum OVX + 15% Dried Plum OVX + 25% Dried Plum for 60 day duration	OVX + 25% Dried Plum: Increased BMD No effect of serum IGF-1, ALP, TRAP
Arjmandi *et al.*, 2010 [46]	SHAM and OVX 3 month old female rats	7 groups: Sham Control OVX-Control OVX +2% FOS OVX +5% FOS + 7.5% Dried Plum OVX + 2% FOS + 5% Dried Plum OVX + 2% FOS + 2% Dried Plum polyphenol OVX + 2% FOS + 7.5% Dried Plum juice	OVX + 5% FOS + 7.5% dried plum showed greatest effect of increased femur BMD
Franklin *et al.*, 2006 [47]	SHAM and ORX 6 month old rats	5 groups: Sham-Control ORX-control ORX + 5% Dried Plum ORX + 15% Dried Plum ORX + 25% Dried Plum for 90 days	ORX + 15%/25% Dried Plum group increased BMD All doses increased serum IGF-1 All doses decreased OPG and RANKL mRNA expression in tibia

ALP: alkaline phosphatase; IGF-1: insulin like growth factor; mRNA: messenger RNA; OVX: ovariectomized; BMD: bone mineral density; TRAP: tartrate resistant acid phosphatase; FOS: fructooligosaccharids; OPG: osteoprotegerin; TNF-α: tumor necrosis factor alpha; ORX: orchidectomized.

**Table 3:** Summary of berry studies on bone health.

First author, year [ref.]	Study Design	Measurements/Treatments	Results
Tanabe *et al.*, 2011 [61]	Human bone marrow cells (pre-osteoclastic)	10, 25, 50, 100 µg/mL cranberry extract 4 d duration	Decreased rate of bone degradation by inhibition of RANKL dependent osteoclasts
Villareal *et al.*, 2007 [50]	SHAM and ORX 1 year old male rats	4 groups: SHAM Control ORX Control ORX + 27% cranberry juice ORX + 45% cranberry juice for 4 months	No effect on bone health
Bickford *et al.*, 2006 [62]	Human bone marrow cells (CD34+ or CD133)	500 ng/mL blueberry extract 72 h duration	Increased proliferation of human bone marrow cells Decreased TRAP staining and RANKL-dependent osteoclast numbers
Devareddy *et al.*, 2008 [56]	SHAM and OVX 6-month old female rats	3 groups: SHAM Control OVX-Control OVX + 5% blueberry for 100 day duration	OVX + 5% blueberry group increased whole body BMD and serum ALP
Chen *et al.*, 2010 [63]	Sprague-Dawley male/female rats; 20 days old (*n* = 20)	2 groups: Control 10% blueberry for 40 day duration	Blueberry: Increases in bone mass, BMD, BMC, Associated with increases in osteoblast number and decreased osteoclast number
Zhang *et al.*, 2011 [58]	Sprague-Dawley female rats, 20 days old	2 groups: Control 10% blueberry diet fed rats only between postnatal day 20 and postnatal day 34	Early blueberry supplementation prevented osteoblast senescence and adult bone loss Blueberry: increased levels of trabecular bone volume, osteoblast number and bone formation rate. Higher osteocalcin levels

OVX: ovariectomized; BMD: bone mineral density; ALP: alkaline phosphatase; TRAP: tartrate resistant acid phosphatase; ORX: orchiodectomy; BMC: bone mineral content.

It should be noted that although berries are a natural source of anthocyanins, some berries are a good source of vitamin C [64]. Vitamin C has demonstrated positive effects on reduced bone resorption and enhanced bone health [65] and as a result may be a confounding factor in attributing anthocyanins only to the preventive effects of osteoporosis. However, when Chen *et al.* [63] isolated polyphenol derived phenolic acids from blueberries and treated ST2 cells, they found upregulation in osteoblast differentiation and osteocalcin production mimicking the results in the blueberry fed rats. This mechanism is further explored in the next section. These results attribute the high bone mass observed in the blueberry fed rats to those phenolic acids [63]. This study is unique and more evidence is needed to determine if anthocyanins are solely responsible, so the possibility exists that both could contribute to its bone prevention qualities. 

From the evidence shown, there is a suggested effect of berry supplementation and overall bone health. However, the mechanism as to how needs to be further explored. Antioxidants, in general, may prevent bone disorder by involvement in anti-oxidative and anti-inflammatory mechanisms. 

## 5. Anti-Oxidative and Anti-Inflammatory Effects of Berries

Phenolic compounds, mainly flavonoids, have a demonstrated role in bone metabolism [17]; however, the mechanisms are not fully understood. Polyphenols may work through the regulation of osteoblasts and osteoclasts [66,67]. Shen *et al.* [18] proposed polyphenols to affect bone through the upregulation of osteoblastogenesis and downregulation of osteoclastogenesis through a variety of mechanisms. These include upregulating runt related transcription factor-2 (Runx2), osteocalcin, the canonical Wnt signaling pathway, β-catenin, and IGF-1, and downregulating RANKL, TRAP, and several matrix metalloproteinases (MMPs) [18]. The canonical Wnt signaling pathway includes a series of growth factors and cascading phosphorylation signals regulated by the protein β-catenin to increase transcription of genes responsible for osteoblast proliferation and differentiation [10]. β-Catenin synergizes with bone-morphogenetic protein-2 (BMP-2) to enhance osteoblast differentiation and bone formation in the activation of Runx2 [68]. When oxidative stress is introduced, β-catenin is diverted from the T cell factors to FoxO mediated transcription, attenuating osteoblastogenesis and bone formation [69]. It is predicted that berries influence the activation of the Wnt signaling pathway through the phosphorylation of mitogen activated protein kinase (MAPK) 38 [10] MAPK p38 is key regulator in bone loss and inhibition of osteoclastogenesis [70]. In rats fed a blueberry diet, phosphorylation of MAPK p38 in femur bone tissue was significantly higher than the control fed rats. The blueberry fed rats also exhibited an increase in β-catenin expression, Runx2, and an overall higher bone mass [63]. To further explore this mechanism *in vitro*, ST2 cells were treated with serum from male and female blueberry diet and control fed rats. ST2 cells are murine bone marrow-derived stromal cells known to undergo osteoblastogenesis in response to Wnt. ST2 cells treated with serum from blueberry fed rats exhibited higher amounts of osteoblast differentiation along with increased ALP, osteocalcin gene expression, and mRNA expression of osteoprotegerin. Phosphorylation of MAPK p38 and β-catenin activation was also increased indicating the possible role of the Wnt signaling pathway. To confirm this pathway, the β-catenin gene was silenced in some ST2 cells. When those cells were treated with serum from blueberry fed rats, there was no observed increase in osteoblast differentiation, indicating a connection between berries and the activation of the Wnt/β-catenin pathway [63]*.*

Polyphenols have also been shown to act through the OPG/RANKL/RANK pathway. Osteoprotegerin (OPG), produced by osteoblasts, inhibits osteoclastogenesis by interfering with binding of RANKL and RANK [67]. In the blueberry fed rats, expression of RANKL mRNA, an osteoclast differentiation marker, was decreased in isolated femur. Osteoclastogenesis was also impaired in these rats. Blueberry supplemented diet in rats was associated with increased osteoblast and decreased osteoclast number *in vivo* [63]. Further supporting this berry hypothesis, cranberry proanthocyanidins have been shown to inhibit osteoclast formation, impair cell maturation, and decrease bone resorption in human pre-osteoclastic cells isolated from human bone marrow [61]. 

At the transcriptional level, flavonoids affect expression of NF-κB in the MAPK pathway [67]. NF-κB controls genes involved in the inflammatory process. During times of oxidative stress NF-κB is increased [67]. The polyphenol action on osteoblasts counteracts the effects of osteoclasts due to oxidative stress. Cyanidin is a potent antioxidant, having been shown to reduce oxidative stress *in vitro* [71] and *in vivo* [72]. Blackberries, which are rich in cyanidins, demonstrate anti-inflammatory properties by inhibiting the release of IL-12 in murine bone marrow derived dendritic cells [73], providing evidence of the anti-inflammatory effect of cyanidin, which may account for the effect of anthocyanins on bone. Blueberry has also been shown to increase proliferation of human bone marrow cells that are also known to be osteoblast progenitor cells [62]. Although limited, evidence does suggest a positive effect of berry consumption on bone health by increasing osteoblast differentiation.

## 6. Conclusions

The natural process of aging causes the body to become more susceptible to oxidative damage. Oxidative stress has shown to increase bone turnover by causing an imbalance between the cells responsible for bone formation and resorption. Antioxidants are known to inhibit and/or ameliorate the effects of oxidative stress. Majority of the human studies highlighted in this review, have demonstrated strong evidence of associations between polyphenol intake, reduced fracture risk, increased bone formation markers, and increased BMD [19,29,39,40,42,43,46]. Animal and cell studies have also reported a positive association of polyphenol intake and bone health [39,40,41,65,66]. Berries, which are naturally rich in anthocyanins have been associated with increased ability to reduce oxidative stress by scavenging free radicals. Evidence provided in the previous studies showed cranberry and blueberry consumption to prevent and ameliorate bone loss [56,58,61,62,63]. Anthocyanins are hypothesized to upregulate osteoblast production through the Wnt signaling pathway, activating MAPK p38, therefore promoting bone formation [18,63]. Polyphenols also seem to inhibit osteoclast formation by inducing secretion of OPG and decreasing RANKL activation of osteoclastogenesis. At the transcription level, they stimulate expression of NF-κB, therefore reducing inflammatory responses [67]. Thus, berry consumption may be a good dietary strategy to lessen the effects of age-related bone loss and decrease the risk of osteoporosis but further studies are warranted to confirm. The studies reviewed do suggest a possible effect and support the need for further study on the underlying mechanisms by which berry consumption in human reduces bone loss caused by aging. 

## Abbreviations

ALP: alkaline phosphatase activity
AOPP: advanced oxidation protein products
AP-1: activator protein-1
BMD: bone mineral density
BMP-2: bone-morphogenetic protein-2
CAM: complementary and alternative medicine
ERK: extracellular signal-regulated kinase
FFQ: food frequency questionnaire
GPx: glutathione peroxidase
IGF-1: Insulin-like growth factor
IL-1: interluekin-1
IL-2: interleukin-2
IL-6: interleukin-6
IL-15: interleukin-15
MAPK: mitogen-activated protein kinase
M-CSF: macrophage colony stimulating factor
MDA: malondialdehyde
MMPs: matrix metalloproteinases
mRNA: messenger RNA
NF-κB: nuclear factor kappa-light-chain-enhancer of activated B cells
OPG: osteoprotegerin
ORX: orchidectomized
OVX: ovariectomized
RANKL: receptor activator of nuclear factor kappa-B ligand
RBC: red blood cell
ROS: reactive oxygen species
Runx2: runt related transcription factor-2
SERM: selective estrogen receptor modulators
SOD: superoxide dismutase
TNF-α: tumor necrosis factor alpha
TRAP: tartrate resistant acid phosphatase

## References

[1] Reginster J.Y., Burlet N. (2006). Osteoporosis: A still increasing prevalence. Bone.

[2] Becker D.J., Kilgore M.L., Morrisey M.A. (2010). The societal burden of osteoporosis. Curr. Rheumatol. Rep..

[3] Burge R., Dawson-Hughes B., Solomon D.H., Wong J.B., King A., Tosteson A. (2007). Incidence and economic burden of osteoporosis-related fractures in the united states, 2005–2025. J. Bone Miner. Res..

[4] Lewiecki E.M. (2011). In the clinic. Osteoporosis. Ann. Intern. Med..

[5] Costa A.G., Wyman A., Siris E.S., Watts N.B., Silverman S., Saag K.G., Roux C., Rossini M., Pfeilschifter J., Nieves J.W. (2013). When, where and how osteoporosis-associated fractures occur: An analysis from the global longitudinal study of osteoporosis in women (glow). PLoS One.

[6] Cauley J.A. (2013). Public health impact of osteoporosis. J. Gerontol. Ser. A Biol. Sci. Med. Sci..

[7] Clarke B.L., Khosla S. (2010). Physiology of bone loss. Radiol. Clin. N. Am..

[8] Lean J.M., Davies J.T., Fuller K., Jagger C.J., Kirstein B., Partington G.A., Urry Z.L., Chambers T.J. (2003). A crucial role for thiol antioxidants in estrogen-deficiency bone loss. J. Clin. Investig..

[9] Chen F., Ouyang Y., Ye T., Ni B., Chen A. (2014). Estrogen inhibits rankl-induced osteoclastic differentiation by increasing the expression of trpv5 channel. J. Cell. Biochem..

[10] Weaver C.M., Alekel D.L., Ward W.E., Ronis M.J. (2012). Flavonoid intake and bone health. J. Nutr. Gerontol. Geriatr..

[11] Zhang Y.B., Zhong Z.M., Hou G., Jiang H., Chen J.T. (2011). Involvement of oxidative stress in age-related bone loss. J. Surg. Res..

[12] (2010). Management of osteoporosis in postmenopausal women: 2010 position statement of the north american menopause society. Menopause.

[13] Singer A., Grauer A. (2010). Denosumab for the management of postmenopausal osteoporosis. Postgrad. Med..

[14] Yang D., Li J., Yuan Z., Liu X. (2013). Effect of hormone replacement therapy on cardiovascular outcomes: A meta-analysis of randomized controlled trials. PLoS One.

[15] Sanders S., Geraci S.A. (2013). Osteoporosis in postmenopausal women: Considerations in prevention and treatment: (Women’s health series). South. Med. J..

[16] Horcajada M.N., Offord E. (2012). Naturally plant-derived compounds: Role in bone anabolism. Curr. Mol. Pharmacol..

[17] Sacco S.M., Horcajada M.N., Offord E. (2013). Phytonutrients for bone health during ageing. Br. J. Clin. Pharmacol..

[18] Shen C.L., von Bergen V., Chyu M.C., Jenkins M.R., Mo H., Chen C.H., Kwun I.S. (2012). Fruits and dietary phytochemicals in bone protection. Nutr. Res..

[19] Hardcastle A.C., Aucott L., Reid D.M., Macdonald H.M. (2011). Associations between dietary flavonoid intakes and bone health in a scottish population. J. Bone Miner. Res..

[20] Paredes-Lopez O., Cervantes-Ceja M.L., Vigna-Perez M., Hernandez-Perez T. (2010). Berries: Improving human health and healthy aging, and promoting quality life—A review. Plant Foods Hum. Nutr..

[21] Krenn L., Steitz M., Schlicht C., Kurth H., Gaedcke F. (2007). Anthocyanin- and proanthocyanidin-rich extracts of berries in food supplements—Analysis with problems. Pharmazie.

[22] Slatnar A., Jakopic J., Stampar F., Veberic R., Jamnik P. (2012). The effect of bioactive compounds on *in vitro* and *in vivo* antioxidant activity of different berry juices. PLoS One.

[23] Aqil F., Gupta A., Munagala R., Jeyabalan J., Kausar H., Sharma R.J., Singh I.P., Gupta R.C. (2012). Antioxidant and antiproliferative activities of anthocyanin/ellagitannin-enriched extracts from *Syzygium cumini* L. (jamun, the indian blackberry). Nutr. Cancer.

[24] Syed F.A., Ng A.C. (2010). The pathophysiology of the aging skeleton. Curr. Osteoporos. Rep..

[25] Almeida M., O’Brien C.A. (2013). Basic biology of skeletal aging: Role of stress response pathways. J. Gerontol. Ser. A Biol. Sci. Med. Sci..

[26] Sanchez-Rodriguez M.A., Ruiz-Ramos M., Correa-Munoz E., Mendoza-Nunez V.M. (2007). Oxidative stress as a risk factor for osteoporosis in elderly mexicans as characterized by antioxidant enzymes. BMC Musculoskelet. Disord..

[27] Almeida M. (2012). Aging mechanisms in bone. BoneKEy Rep..

[28] Filaire E., Toumi H. (2012). Reactive oxygen species and exercise on bone metabolism: Friend or enemy?. Jt. Bone Spine Rev. Rhum..

[29] Garrett I.R., Boyce B.F., Oreffo R.O., Bonewald L., Poser J., Mundy G.R. (1990). Oxygen-derived free radicals stimulate osteoclastic bone resorption in rodent bone *in vitro* and *in vivo*. J. Clin. Investig..

[30] Mody N., Parhami F., Sarafian T.A., Demer L.L. (2001). Oxidative stress modulates osteoblastic differentiation of vascular and bone cells. Free Radic. Biol. Med..

[31] Bai X.C., Lu D., Bai J., Zheng H., Ke Z.Y., Li X.M., Luo S.Q. (2004). Oxidative stress inhibits osteoblastic differentiation of bone cells by erk and nf-kappab. Biochem. Biophys. Res. Commun..

[32] Maggio D., Barabani M., Pierandrei M., Polidori M.C., Catani M., Mecocci P., Senin U., Pacifici R., Cherubini A. (2003). Marked decrease in plasma antioxidants in aged osteoporotic women: Results of a cross-sectional study. J. Clin. Endocrinol. Metab..

[33] Muthusami S., Ramachandran I., Muthusamy B., Vasudevan G., Prabhu V., Subramaniam V., Jagadeesan A., Narasimhan S. (2005). Ovariectomy induces oxidative stress and impairs bone antioxidant system in adult rats. Clin. Chim. Acta.

[34] Sontakke A.N., Tare R.S. (2002). A duality in the roles of reactive oxygen species with respect to bone metabolism. Clin. Chim. Acta.

[35] Nojiri H., Saita Y., Morikawa D., Kobayashi K., Tsuda C., Miyazaki T., Saito M., Marumo K., Yonezawa I., Kaneko K. (2011). Cytoplasmic superoxide causes bone fragility owing to low-turnover osteoporosis and impaired collagen cross-linking. J. Bone Miner. Res..

[36] Suntar I., Akkol E.K. (2013). Beneficial effects of plant sources on the treatment of osteoporosis. Curr. Drug Targets.

[37] Levis S., Lagari V.S. (2012). The role of diet in osteoporosis prevention and management. Curr. Osteoporos. Rep..

[38] Hamidi M., Boucher B.A., Cheung A.M., Beyene J., Shah P.S. (2011). Fruit and vegetable intake and bone health in women aged 45 years and over: A systematic review. Osteoporos. Int..

[39] New S.A., Robins S.P., Campbell M.K., Martin J.C., Garton M.J., Bolton-Smith C., Grubb D.A., Lee S.J., Reid D.M. (2000). Dietary influences on bone mass and bone metabolism: Further evidence of a positive link between fruit and vegetable consumption and bone health?. Am. J. Clin. Nutr..

[40] Langsetmo L., Hanley D.A., Prior J.C., Barr S.I., Anastassiades T., Towheed T., Goltzman D., Morin S., Poliquin S., Kreiger N. (2011). Dietary patterns and incident low-trauma fractures in postmenopausal women and men aged ≥50 y: A population-based cohort study. Am. J. Clin. Nutr..

[41] McTiernan A., Wactawski-Wende J., Wu L., Rodabough R.J., Watts N.B., Tylavsky F., Freeman R., Hendrix S., Jackson R. (2009). Low-fat, increased fruit, vegetable, and grain dietary pattern, fractures, and bone mineral density: The women’s health initiative dietary modification trial. Am. J. Clin. Nutr..

[42] Welch A., MacGregor A., Jennings A., Fairweather-Tait S., Spector T., Cassidy A. (2012). Habitual flavonoid intakes are positively associated with bone mineral density in women. J. Bone Miner. Res..

[43] Hooshmand S., Chai S.C., Saadat R.L., Payton M.E., Brummel-Smith K., Arjmandi B.H. (2011). Comparative effects of dried plum and dried apple on bone in postmenopausal women. Br. J. Nutr..

[44] Arjmandi B.H., Khalil D.A., Lucas E.A., Georgis A., Stoecker B.J., Hardin C., Payton M.E., Wild R.A. (2002). Dried plums improve indices of bone formation in postmenopausal women. J. Women’s Health Gend. Based Med..

[45] Deyhim F., Stoecker B.J., Brusewitz G.H., Devareddy L., Arjmandi B.H. (2005). Dried plum reverses bone loss in an osteopenic rat model of osteoporosis. Menopause.

[46] Arjmandi B.H., Johnson C.D., Campbell S.C., Hooshmand S., Chai S.C., Akhter M.P. (2010). Combining fructooligosaccharide and dried plum has the greatest effect on restoring bone mineral density among select functional foods and bioactive compounds. J. Med. Food.

[47] Franklin M., Bu S.Y., Lerner M.R., Lancaster E.A., Bellmer D., Marlow D., Lightfoot S.A., Arjmandi B.H., Brackett D.J., Lucas E.A. (2006). Dried plum prevents bone loss in a male osteoporosis model via igf-i and the rank pathway. Bone.

[48] Halloran B.P., Wronski T.J., VonHerzen D.C., Chu V., Xia X., Pingel J.E., Williams A.A., Smith B.J. (2010). Dietary dried plum increases bone mass in adult and aged male mice. J. Nutr..

[49] Huang W.Y., Zhang H.C., Liu W.X., Li C.Y. (2012). Survey of antioxidant capacity and phenolic composition of blueberry, blackberry, and strawberry in nanjing. J. Zhejiang Univ. Sci. B.

[50] Villarreal A., Stoecker B.J., Garcia C., Garcia K., Rios R., Gonzales C., Mandadi K., Faraji B., Patil B.S., Deyhim F. (2007). Cranberry juice improved antioxidant status without affecting bone quality in orchidectomized male rats. Phytomedicine.

[51] Jakesevic M., Aaby K., Borge G.I., Jeppsson B., Ahrne S., Molin G. (2011). Antioxidative protection of dietary bilberry, chokeberry and lactobacillus plantarum heal19 in mice subjected to intestinal oxidative stress by ischemia-reperfusion. BMC Complement. Altern. Med..

[52] Karlsen A., Paur I., Bohn S.K., Sakhi A.K., Borge G.I., Serafini M., Erlund I., Laake P., Tonstad S., Blomhoff R. (2010). Bilberry juice modulates plasma concentration of NF-kappab related inflammatory markers in subjects at increased risk of CVD. Eur. J. Nutr..

[53] Karlsen A., Retterstol L., Laake P., Paur I., Bohn S.K., Sandvik L., Blomhoff R. (2007). Anthocyanins inhibit nuclear factor-kappab activation in monocytes and reduce plasma concentrations of pro-inflammatory mediators in healthy adults. J. Nutr..

[54] Kim B., Ku C.S., Pham T.X., Park Y., Martin D.A., Xie L., Taheri R., Lee J., Bolling B.W. (2013). Aronia melanocarpa (chokeberry) polyphenol-rich extract improves antioxidant function and reduces total plasma cholesterol in apolipoprotein e knockout mice. Nutr. Res..

[55] Mane C., Loonis M., Juhel C., Dufour C., Malien-Aubert C. (2011). Food grade lingonberry extract: Polyphenolic composition and *in vivo* protective effect against oxidative stress. J. Agric. Food Chem..

[56] Devareddy L., Hooshmand S., Collins J.K., Lucas E.A., Chai S.C., Arjmandi B.H. (2008). Blueberry prevents bone loss in ovariectomized rat model of postmenopausal osteoporosis. J. Nutr. Biochem..

[57] Basu A., Rhone M., Lyons T.J. (2010). Berries: Emerging impact on cardiovascular health. Nutr. Rev..

[58] Zhang J., Lazarenko O.P., Blackburn M.L., Shankar K., Badger T.M., Ronis M.J., Chen J.R. (2011). Feeding blueberry diets in early life prevent senescence of osteoblasts and bone loss in ovariectomized adult female rats. PLoS One.

[59] Bu S.Y., Hunt T.S., Smith B.J. (2009). Dried plum polyphenols attenuate the detrimental effects of TNF-alpha on osteoblast function coincident with up-regulation of runx2, osterix and IGF-i. J. Nutr Biochem..

[60] Bu S.Y., Lerner M., Stoecker B.J., Boldrin E., Brackett D.J., Lucas E.A., Smith B.J. (2008). Dried plum polyphenols inhibit osteoclastogenesis by downregulating nfatc1 and inflammatory mediators. Calcif. Tissue Int..

[61] Tanabe S., Santos J., La V.D., Howell A.B., Grenier D. (2011). A-type cranberry proanthocyanidins inhibit the rankl-dependent differentiation and function of human osteoclasts. Molecules.

[62] Bickford P.C., Tan J., Shytle R.D., Sanberg C.D., El-Badri N., Sanberg P.R. (2006). Nutraceuticals synergistically promote proliferation of human stem cells. Stem Cells Dev..

[63] Chen J.R., Lazarenko O.P., Wu X., Kang J., Blackburn M.L., Shankar K., Badger T.M., Ronis M.J. (2010). Dietary-induced serum phenolic acids promote bone growth via p38 mapk/beta-catenin canonical WNT signaling. J. Bone Miner. Res..

[64] Del Rio D., Borges G., Crozier A. (2010). Berry flavonoids and phenolics: Bioavailability and evidence of protective effects. Br. J. Nutr..

[65] Nieves J.W. (2013). Skeletal effects of nutrients and nutraceuticals, beyond calcium and vitamin D. Osteoporos. Int..

[66] Williams R.J., Spencer J.P., Rice-Evans C. (2004). Flavonoids: Antioxidants or signalling molecules?. Free Radic. Biol. Med..

[67] Trzeciakiewicz A., Habauzit V., Horcajada M.N. (2009). When nutrition interacts with osteoblast function: Molecular mechanisms of polyphenols. Nutr. Res. Rev..

[68] Mbalaviele G., Sheikh S., Stains J.P., Salazar V.S., Cheng S.L., Chen D., Civitelli R. (2005). Beta-cateninand bmp-2 synergize to promote osteoblast differentiation and new bone formation. J. Cell. Biochem..

[69] Almeida M., Han L., Martin-Millan M., O’Brien C.A., Manolagas S.C. (2007). Oxidative stress antagonizes wnt signaling in osteoblast precursors by diverting beta-catenin from t cell factor- to forkhead box o-mediated transcription. J. Biol. Chem..

[70] Boyle D.L., Hammaker D., Edgar M., Zaiss M.M., Teufel S., David J.P., Schett G., Firestein G.S. (2014). Differential roles of mapk kinases mkk3 and mkk6 in osteoclastogenesis and bone loss. PLoS One.

[71] Choi M.J., Kim B.K., Park K.Y., Yokozawa T., Song Y.O., Cho E.J. (2010). Anti-aging effects of cyanidin under a stress-induced premature senescence cellular system. Biol. Pharm. Bull..

[72] Tsuda T., Horio F., Osawa T. (2000). The role of anthocyanins as an antioxidant under oxidative stress in rats. BioFactors.

[73] Dai J., Patel J.D., Mumper R.J. (2007). Characterization of blackberry extract and its antiproliferative and anti-inflammatory properties. J. Med. Food.

